# Cross-Cultural Adaptation and Measurement Properties of the Expectations for Treatment Scale (ETS) for Greek-Speaking Patients

**DOI:** 10.7759/cureus.46457

**Published:** 2023-10-04

**Authors:** Stefanos Karanasios, Nektarios Martzoukos, Nikolaos Zampetakis, Danai Paleta, Thomas Sampsonis, Ioannis Vasilogeorgis, George Gioftsos

**Affiliations:** 1 Physiotherapy, University of West Attica, Athens, GRC; 2 Physiotherapy, Hellenic OMT eDu, Athens, GRC

**Keywords:** validation, translation, musculoskeletal, prognostic factor, treatment expectations

## Abstract

Introduction

Patients’ treatment expectations are key factors influencing the health outcomes in various medical conditions. Using validated measures to capture these expectations has been strongly suggested to improve the prognosis of the health outcome and promote research investigations. The Expectations for Treatment Scale (ETS) is a well-established questionnaire designed to accurately measure treatment expectations in patients with low back pain; however, it is not available in Greek yet. We aimed to translate and cross-culturally adapt the ETS in Greek (ETS-Gr) and evaluate its reliability and validity in a Greek-speaking population with musculoskeletal disorders.

Methods

We followed published recommendations for the translation and cross-cultural adaptation process of the scale. Face and content validity were evaluated using interviews with patients and experts. Internal consistency, test-retest reliability, and measurement error were evaluated in 52 patients with musculoskeletal conditions.

Results

During forward and backward translation minor linguistic discrepancies were detected and effectively adapted for Greek-speaking patients. The ETS-Gr presented a high level of content validity (item content validity index: 0.88-1; and average scale content validity index: 0.90), acceptable internal consistency (Cronbach’s alpha: 0.84), and excellent test-retest reliability (intraclass correlation coefficient: 0.96, 95% confidence interval: 0.93-0.98).

Conclusions

The ETS-Gr is a short, reliable, and valid instrument to measure pre-treatment expectations in patients with musculoskeletal disorders. Future investigations including other medical conditions are required.

## Introduction

The role of patients’ expectations in treatment outcomes has received increasing attention in medical research during the last two decades [1,2]. Patients’ expectations are considered critical predictors of the analgesic outcome across conditions, such as cancer, stroke, musculoskeletal pain (i.e., knee osteoarthritis and low back pain), and port surgery [3-7]. They have been proposed as the driving mechanism of the placebo or nocebo effects that may either enhance healthcare outcomes or increase the occurrence of adverse events [4,5,8]. For example, studies showed that positive expectations for acupuncture or manipulative therapy were related to reduced pain in patients with low back pain [9-11]. Accordingly, negative expectancies were a powerful factor in the development of adverse treatment outcomes, such as nausea from chemotherapy [12,13]. Although patients’ expectations can influence health outcomes, the size of this effect remains unclear and depends on factors such as patient experiences, structure and process of health care, style of communication between therapists and patients, and information provided [14,15].

Despite growing research investigations on patients’ expectations and outcomes, the integration of research findings in the current field is problematic [16]. The most important limitation is the increased heterogeneity of treatment expectation measures concerning the wide variety of the diseases being investigated [14,17]. Hence, questionnaires designed to evaluate treatment expectations for certain pathologies seem inappropriate for other medical conditions [18]. Moreover, other measures have been criticised for their content validity concerning the type or extent of expectations being measured [14,16]. The accurate assessment of pretreatment expectations in musculoskeletal conditions can be a valuable step to accurately predict treatment responses [16,19]. Therefore, using valid and reliable questionnaires is necessary to enhance the subjective evaluation of musculoskeletal patients and provide accurate research and clinical data [19,20].

The Expectation for Treatment Scale (ETS) is a short (five-item) measure developed to capture outcome-related expectations of patients with low back pain (Appendix 1) [19]. Although it was developed using acupuncture as a case example, it can be easily adapted to other clinical situations and treatments [19]. The ETS has shown excellent reliability and validity [19]. The availability of translated and validated expectation scales in different languages is a critical step to facilitate comparisons among patient groups with the same medical condition and promote international research in the same field of interest [21]. However, based on our knowledge, a Greek version of the ETS (ETS-Gr) is not available yet.

We hypothesised that a valid and reliable version of the ETS in Greek will enable the accurate evaluation of treatment expectations as a standardised research and clinical mechanism for a variety of musculoskeletal pathologies. Our study aimed to translate and cross-culturally adapt the English version of the questionnaire into Greek and evaluate its reliability and validity in a cohort of Greek-speaking patients with musculoskeletal pathologies.

## Materials and methods

Cross-cultural adaptation

Our observational cross-sectional study was conducted following published recommendations and guidelines for the translation and cross-cultural adaptation of patient-reported outcome measures [21-23].

Step 1 - Forward translation (FT): The ETS was translated by a professional translator without a medical background and a bilingual physiotherapist (native Greek speaker) with 18 years of experience in musculoskeletal practice. They produced two Greek translations of the scale (FT-1 and FT-2).

Step 2 - Synthesis: The FT-1 and FT-2 were synthesised into one by a research committee consisting of two translators and four bilingual physiotherapists. The members of the committee followed a consensus process. All changes and decisions during the synthesis process were recorded.

Step 3 - Back translation (BT): The consensus version of the FT was independently translated back into English by two other translators (a medical practitioner and an English teacher) who were ‘naïve’ about the content of the scale.

Step 4 - BT review and synthesis: The research committee carefully reviewed the two versions of the BT focusing on semantic, idiomatic, experimental, and conceptual equivalence [22]. All discrepancies were resolved using a consensus process. Minor changes were made and an updated version of the FT was produced.

Step 5 - Validation of translation: We included a formal evaluation of the comparability of language and the similarity of interpretability by five physiotherapy researchers using a 7-point Likert scale (1: extremely comparable/similar, 7: not at all comparable/similar). A mean score >3 in the comparability of language rating and >2.5 in the similarity of interpretability rating required review for possible corrections. We also included a process of cognitive debriefing by recruiting eight healthy individuals (four men and four women with a mean age of 26.1 years). The participants were fluent in Greek and were inquired for cognitive equivalence of the questionnaire.

Step 6 - Review and finalisation: All comments provided by the responders were carefully considered by the research committee and some necessary changes were made before the production of the pre-final version of the questionnaire.

Step 7 - Pretesting: The content validity of each item of the questionnaire was evaluated by nine physiotherapy researchers using a Likert scale from 1 (not relevant and clear) to 4 (highly relevant and clear). Moreover, 10 patients participated in a pilot analysis as volunteers to facilitate evaluating the pre-final version of the scale. The patients completed the scale and were subsequently interviewed by a member of the research committee. The interviews focused on the comprehensibility of the items, wording, terminology, instructions, clarity of the responses, comprehensiveness, and relevance of the questionnaire to their condition. Then, the final version of the ETS-Gr was produced.

Patients and procedures

Patients were recruited from a physiotherapy clinic in Athens (Physio-Kifisia) between the 1st of May and the 30th of August 2023. All participants signed a written informed consent before participation in the study. Ethical approval was granted by the University of West Attica Ethics Committee, Greece (ID: 40713/25-04-2023, date: 25-04-2023). The inclusion criteria were pain of musculoskeletal origin (i.e., knee osteoarthritis, neck or back pain, upper or lower limb tendinopathies) as diagnosed by attending a medical practitioner, pain lasting longer than 15 days, older than 18 years of age, and fluency in Greek. The exclusion criteria were rheumatoid arthritis or other chronic inflammatory diseases, recent fractures (<3 months), tumours, neurological disorders, cognitive disability, unable to read Greek, and being younger than 18 years old.

On the first visit, demographic characteristics, such as age, sex, body mass index (BMI), diagnosis, and duration of symptoms, were recorded. Then, all participants filled out the Greek version of the ETS. To assess the test-retest reliability of the questionnaire, a second copy was administered between five and seven days after their first visit. After each completion, the questionnaires were checked for missing items, and full responses were ensured.

Instrument

The ETS is a brief five-item questionnaire designed to capture outcome-related expectations (18). The scale is available in German, French, and English. Each of the five items of the ETS is rated on a four-point scale, ranging from 1 (partially disagree) to 4 (definitely agree). The total score fluctuates from 5 to 20 points with the higher values indicating higher expectations about treatment. It has presented an acceptable internal consistency (Cronbach’s α = 0.77), excellent test-retest reliability (intraclass correlation coefficient (ICC) = 0.87), and high convergent validity compared to another treatment-specific expectation measure (r>0.90) (18).

Statistical analysis

Based on COnsensus-based Standards for the selection of health status Measurement INstruments (COSMIN) recommendations for the evaluation of test-retest reliability and measurement error, a minimum sample size of 50 participants was considered adequate [24,25]. Data were analysed with Statistical Product and Service Solutions (SPSS) (version 25; IBM SPSS Statistics for Windows, Armonk, NY). The level of statistical significance was set at p < 0.05. We have used descriptive statistics to determine the mean (± standard deviation (SD)) or percentage for participants’ demographic characteristics (i.e., age, sex, BMI, duration of symptoms, and ETS scores). The Shapiro-Wilk test and Q-Q plots were used to ensure that the data were normally distributed.

The content validity index (CVI) was determined by computing the items rated with 3 or 4 (relevant and clear items) divided by the number of raters (nine experts in the field). The item-CVI was considered acceptable for values > 0.78 and the scale-CVI for values > 0.80 [26].

We have calculated Cronbach’s alpha to evaluate the internal consistency of the ETS-Gr. Based on the literature, Cronbach’s alpha values between 0.70 and 0.95 suggest a good internal consistency [23]. The ICC2,1 (two-way random model, absolute agreement) was used to determine test-retest reliability with a 95% confidence interval (CI). The ICC was considered poor, fair, and excellent for values <0.4, 0.4-0.75, and >0.75, respectively [27].

The standard error of measurement (SEM) [$SEM= SD\times \sqrt{1-test retest reliability coefficient}$] and the minimal detectable change (MDC) [$MDC95= 1.96\times \sqrt{2}\times SEM$] were calculated to determine absolute reliability. The Bland-Altman plot with a scatter plot of differences and 95% limits of agreement were used to visualise the agreement between the scores of the repeated administrations [28].

The floor and ceiling effects of the questionnaire were determined if > 15% of the participants obtained the lowest (5 points) or the highest (20 points) possible score [29]. The time needed to complete the ETS-Gr was recorded as well.

## Results

Minor linguistic discrepancies were found during the FT and BT of the ETS (Table 1). Subsequent re-evaluation of the comparability of language and similarity of interpretability presented no further issues. Nine experts and 10 patients (six men, mean age: 32.1 years) with various musculoskeletal problems including four with low back pain, four with neck pain, and two with knee osteoarthritis were interviewed for comprehensibility. No issues were reported about wording, instructions, and clarity of the responses, resulting in the final version of the ETS-Gr. Moreover, all participants reported that the questionnaire was relevant to the treatment expectations of their condition.

**Table 1:** List of items that were adjusted during cross-cultural translation

Items requiring adjustment	Rationale	Solutions
Complaints	The literal translation was not considered appropriate.	Instead, the word ‘symptoms’ translated into Greek was used.
Imputation procedures	The literal translation was not considered appropriate.	Instead, the expression ‘attribution procedures’ translated into Greek was used.

Participants 

A total of 52 patients (28 women and 24 men) with various musculoskeletal pathologies and a mean age (±SD) of 43.07 (±19.6) years participated in the study. Table 2 shows participants’ demographic characteristics. The responders required three to four minutes to complete the questionnaire.

**Table 2:** Demographic and clinical characteristics of participants (N = 52)

Characteristic	Mean ±SD (range) or No (percentage)
Age (years)	43.7 ±19.6 (18-85)
Sex	
Men	24 (46.2%)
Women	28 (53.8%)
Diagnosis	
Rotator-cuff related pain	15
Non-specific mechanical neck pain	10
Non-specific mechanical low back pain	9
Anterior knee pain	8
Hamstring tear	3
Achilles tendinopathy	3
Lateral elbow tendinopathy	2
Plantar fasciopathy	2
Height (cm)	170.2 ±9 (150-191)
Weight (kg)	75.7 ±17.5 (44-130)
ETS-Gr	15.3 ±3.03 (6-20)
Item 1	3.33 ±0.7
Item 2	2.55 ±0.9
Item 3	3.03 ±0.7
Item 4	3.11 ±0.6
Item 5	3.30 ±0.7
Abbreviations: ETS-Gr, Expectation for Treatment Scale – Greece; N, sample; SD, standard deviation; cm, centimeters; kg, kilograms; No, number	

Validity

The item-CVI was 1.00 for Item 4 and 0.88 for Items 1, 2, 3, and 5. The average scale-CVI was 0.90, suggesting excellent content validity. No floor or ceiling effects were reported.

Reliability

The internal consistency of the ETS-Gr was acceptable, with Cronbach’s alpha of 0.84. The test-retest reliability was excellent, reporting an ICC of 0.96 (95% CI = 0.93-0.98). The SEM and MDC95 were 0.12 and 0.33 points (scales 0 to 20), respectively (Table 3).

**Table 3:** Test-rest reliability and internal consistency of WORC-GR Abbreviations: ETS-Gr, Expectation for Treatment Scale – Greece; ICC, intraclass correlation coefficient; SEM, standard error of measurement; MDC, minimal detectable change; CI, confidence interval

	Cronbach’s alpha	ICC (95% CI)	SEM % (Points)	MDC_95 _% (Points)
ETS-Gr	0.84	0.96 (0.93-0.98)	0.6 (0.12)	1.67 (0.33)

**Figure 1:**
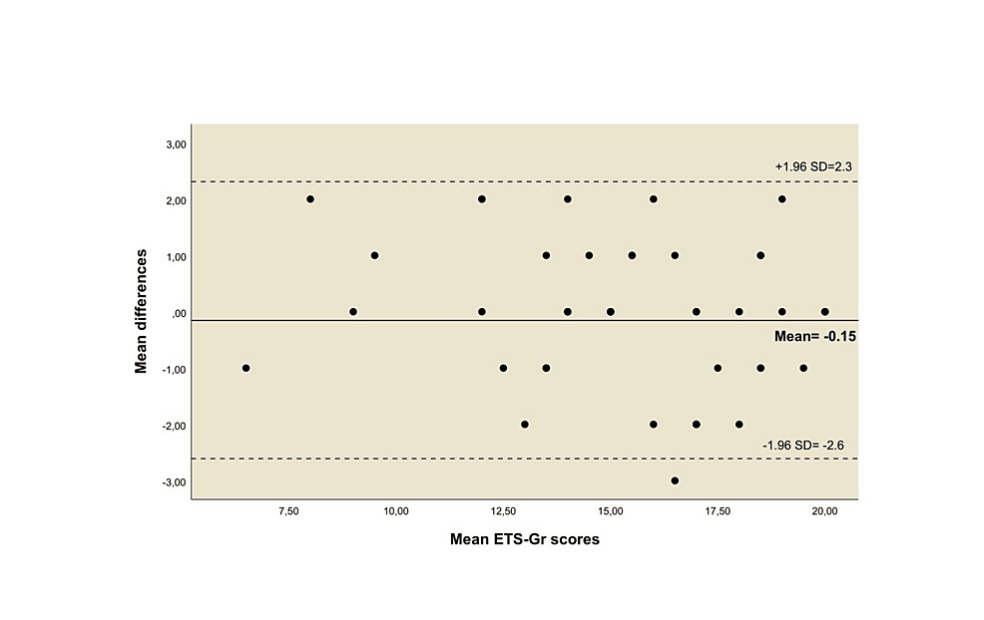
Bland-Altman plot of the agreement between the test and retest scores of the ETS-Gr

Figure 1 shows the Bland-Altman plot indicating no systematic bias or proportional error with a strong agreement and minimal within-subject variation between the scores of the two administrations. The mean difference was small (±SD) of -0.15 (±1.25), the limits of agreement ranged between -2.6 and 2.3, and the t-score was not statistically significant (t=-0.882, p = 0.382). Only one score was out of the limits of the agreement.

## Discussion

The ETS is an important instrument in research and clinical settings to capture patients’ pretreatment expectations that may predict healthcare outcomes [9,19]. International research is rapidly developing, with a large number of articles published every year in the current field [14,30]. Thus, having a validated ETS for Greek-speaking patients can support similar clinical studies in the current population. According to the present study findings, the ETS was successfully translated and cross-culturally adapted into Greek. The ETS-Gr showed adequate face and content validity. Additionally, it presented excellent internal consistency, test-test reliability, and acceptable measurement error.

The translation and cross-cultural adaptation of the ETS were derived from suggested and recognised guidelines [22,23,29]. Although most relevant studies include five basic steps in their methodology (i.e., FT, synthesis, BT, review by an expert committee, and pretesting) [22], we adopted a recently proposed approach using a merged methodology to enhance the rigour of the process [29,31,32]. Specifically, we included the assessment of the comparability of language, the similarity of interpretability, and cognitive equivalence for the cross-cultural adaptation of the ETS by a mixed panel of experts, including translators, content experts, bilingual clinicians, and native speakers [29,31,32]. Minor linguistic discrepancies between the English and Greek versions were detected during the translation process and were appropriately resolved by the research committee.

The content validation process is another critical step in the development of an instrument, especially when it is used to measure health outcomes or guide clinical decision-making [33]. During the adaptation of the ETS in Greek, we evaluated the comprehensibility, relevance, and comprehensiveness of the questionnaire using semi-structured interviews. Another important factor in the process was the calculation of the item-CVI and scale-CVI as translated by Polit et al. into values of a modified kappa statistic [26]. Our analysis suggested the simplicity and clearness of the ETS-Gr, while both item-CVI and average scale-CVI exceeded the minimum cutoff points of 0.78 and 0.90, respectively. Overall, the ETS-Gr proved to be a self-administered, comprehensible, and relevant tool for patients’ treatment expectations in musculoskeletal conditions.

Although an optimal value of Cronbach’s alpha is debatable, values lower than 0.70 may indicate item heterogeneity, while values higher than 0.95 may suggest item redundancy [23]. Internal consistency of the ETS-Gr was good to excellent (Cronbach’s alpha = 0.84) with no item redundancy and consistency with the English version (Cronbach’s alpha = 0.90) [19].

The Greek version of the ETS presented excellent test-retest reliability (ICC = 0.96), which was higher compared to the English version of the questionnaire (ICC = 0.86) [19]. This difference may be attributed to the time between the two administrations in our study (four to seven days) compared to the time used in the English version (one week). The evaluation of instrument repeatability requires the selection of an appropriate time interval between measurements that will be simultaneously long enough to reduce the possibility of recall bias and short enough to ensure that the patient's condition has not changed [34]. Two weeks is considered the most appropriate time interval for test-retest reliability measures [35]. However, such a long period between administrations in a clinical setting can be unfeasible and underlies a high risk of attrition bias. Hence, in the present study, we selected a short time to ensure the stability of the patient’s treatment expectations, limit the risk of recall bias, and reduce the drop-out rate.

Although floor and ceiling effects are important psychometric properties that can significantly influence the validity, reliability, and responsiveness of a scale, they are usually underestimated or poorly reported [36-38]. Based on our findings, the ETS-Gr reported no floor and ceiling effects, suggesting that it can be a valid measure in a Greek-speaking population with musculoskeletal conditions.

The present study findings should be interpreted in light of some limitations. First, using a single-centre sample may underlie a potential risk of bias for the evaluation of the psychometric properties of a new scale. Second, the short time interval (four to seven days) between the administrations may have increased the risk of recall bias in our study [35]. Third, our sample included only patients with musculoskeletal conditions, which may limit the generalisability of our results to other patient groups. Therefore, further research should evaluate the psychometric properties of the ETS-Gr in patients with various medical and psychological conditions where treatment expectations are critical predictors of health outcomes, such as obesity, depression, and cancer. Fourth, although convergent validity is a critical psychometric property that measures how closely a new scale is related to other variables and other measures of the same construct [23,34], our study did not include this evaluation. Further evaluation of the convergent validity of the ETS-Gr is necessary for the extended clinical use of the scale.

## Conclusions

The ETS was successfully translated into Greek, presenting a comprehensible and valid measure of treatment expectations in patients with musculoskeletal conditions. It presented an acceptable internal consistency, excellent test-retest reliability, and a small measurement error. The ETS-Gr provides a trustworthy and flexible tool in clinical studies that can further be used to investigate the relationship between treatment-related expectations and health outcomes. Future studies evaluating the psychometric properties of the scale in other medical conditions are essential.

## Appendices

**Table 4:** Εxpectation for treatment scale (ETS)

English version. There are several statements below that capture your expectations about the treatment. Please indicate to what extent these statements apply to you personally. There are no right or wrong answers. We are only interested in your current personal thoughts. Please select one response for each statement.			
1. I expect the treatment will help me to cope with my complaints.			
partially disagree	partially agree	agree	definitely agree
O	O	O	O
2. I expect the treatment will make my complaints disappear.			
partially disagree	partially agree	agree	definitely agree
O	O	O	O
3. I expect the treatment will improve my energy.			
partially disagree	partially agree	agree	definitely agree
O	O	O	O
4. I expect the treatment will improve my physical performance.			
partially disagree	partially disagree	partially disagree	partially disagree
O	O	O	O
5. I expect that after the treatment, my complaints will be considerably better.			
partially disagree	partially disagree	partially disagree	partially disagree
O	O	O	O
